# Can Preoperative Blood Inflammatory Biomarkers Predict Early Dental Implant Outcomes in Systemically Healthy Patients?

**DOI:** 10.3390/bioengineering12111208

**Published:** 2025-11-05

**Authors:** Elena-Raluca Baciu, Cezara Andreea Onică, Gabriela Luminița Gelețu, Neculai Onică, Bogdan Florin Toma, Alexandra Cornelia Teodorescu, Costin Iulian Lupu, Alice Murariu

**Affiliations:** 1 Faculty of Dental Medicine, Grigore T. Popa University of Medicine and Pharmacy, 700115 Iasi, Romania; elena.baciu@umfiasi.ro (E.-R.B.); gabriela.geletu@umfiasi.ro (G.L.G.); onica_neculai@d.umfiasi.ro (N.O.); cornelia.teodorescu@umfiasi.ro (A.C.T.); iulian.lupu@umfiasi.ro (C.I.L.); alice.murariu@umfiasi.ro (A.M.); 2 Faculty of Medicine, Grigore T. Popa University of Medicine and Pharmacy, 700115 Iasi, Romania; bogdan.f.toma@umfiasi.ro

**Keywords:** inflammatory biomarkers, C-reactive protein, osseointegration, dental implants

## Abstract

The aim of this study was to assess whether baseline the neutrophil-to-lymphocyte ratio (NLR), the platelet-to-lymphocyte ratio (PLR), the systemic immune-inflammation index (SII), and C-reactive protein (CRP) could predict postoperative outcomes in systemically healthy patients receiving dental implants. A retrospective analysis of 116 systemically healthy adults receiving dental implants was conducted. To minimise confounding, individuals over 50 years old, smokers, and those with systemic diseases, stage III/IV periodontitis, or current medication use were excluded. Periodontal status was classified as clinically healthy or stable. Baseline CRP and complete blood count-derived indices (NLR, PLR, SII) were recorded preoperatively. The primary outcome was osseointegration (proper versus impaired). Implant success was 95.7% (n = 111), with early implant failure occurring in 4.3% (n = 5). Females exhibited higher PLR values than males (*p* = 0.041), and SII was higher in periodontally stable patients compared to clinically healthy ones (*p* = 0.036). In systemically healthy patients, routine preoperative screening based on NLR, PLR, and CRP did not improve prediction of early implant failure, whereas SII demonstrated good, statistically significant discrimination (*p* = 0.015). These findings emphasise the need for further research to clarify the predictive value of blood inflammatory biomarkers.

## 1. Introduction

Dental implant therapy consistently demonstrates high success rates when performed following established surgical and prosthetic guidelines, supported by the extensive scientific literature [1,2,3]. Implant failure is classified as either biological or prosthetic, with biological failure indicating inadequate osseointegration [4,5,6,7]. Early failure occurs before or at the time of healing-abutment placement and is primarily caused by poor integration [8]. Osseointegration is defined as the direct structural and functional connection between living, organised bone and the surface of a load-bearing implant, without the interposition of soft tissue [9]. First described by Brånemark, it provides a stable anchorage mechanism whereby the implant becomes permanently incorporated into bone, enabling long-term prosthetic support [10,11]. Clinically, an implant is considered osseointegrated when there is no progressive relative movement between the implant and the surrounding bone [9]. Neutrophils and macrophages participate in the early healing response [12,13]. After implant placement, they rapidly gather and release mediators that recruit and promote the proliferation and differentiation of osteoprogenitor cells, supporting contact osteogenesis at the surface and distance osteogenesis in the surrounding bone [14,15]. The success of this process is influenced by patient factors (age, systemic conditions, bone and soft tissue quality and vascularity, inflammatory status, medications, smoking, and nutritional status), implant factors (material composition, implant design, surface topography, energy, and wettability), and the surgical technique [16,17,18]. Blood inflammatory biomarkers such as the neutrophil-to-lymphocyte ratio (NLR), platelet-to-lymphocyte ratio (PLR), and systemic immune-inflammation index (SII) have recently gained increasing attention as valuable indicators of systemic inflammation, complementing traditional laboratory markers like C-reactive protein (CRP), which have long been used in clinical practice.

Neutrophils (Ns) are vital cells in the innate immune system that assist early bone formation by creating a temporary, fibronectin-rich matrix (“emergency extracellular matrix”) at injury sites that attracts stromal cells [19]. Within three days, fibronectin accumulates in the haematoma, and stromal cells fill the gaps, depositing type I collagen that promotes new bone growth [19]. When post-traumatic inflammation is severe, neutrophils become overactivated and dysfunctional, releasing high levels of cytokines and causing substantial neutrophil extracellular trap formation, which exacerbates tissue damage and negatively impacts nearby healthy tissues [13]. Lymphocytes (Ls), including B and T cell subsets, participate in both innate and adaptive immune responses. Platelets (PLTs), or thrombocytes, mainly function in haemostasis and vascular regulation, with their levels increasing during acute inflammatory reactions. In this context, PLR has been proposed as an indicator linking inflammatory activity with thrombotic risk. The SII, defined as (platelet count × neutrophil count)/lymphocyte count, is a recently proposed index for evaluating systemic inflammatory activity. Elevated SII values have been observed in various pathological conditions, including cardiovascular and rheumatological disorders, osteoporosis, several malignancies, and metabolic diseases [20].

C-reactive protein (CRP) plays a key role in the innate immune response by binding to damaged cells and pathogens, activating the complement system and promoting phagocytosis. While it can promote inflammation and tissue damage, CRP also exhibits anti-inflammatory effects, acting as both a stimulator and regulator of inflammation. In healthy individuals, CRP is typically present at low concentrations, averaging approximately 0.8 mg/L (normal CRP levels vary between populations, with mean values between 1.0 and 3.0 mg/L, and, for healthy, young subjects, less than 1.5 mg/L). However, as a sensitive marker of the acute phase response, CRP levels can rise rapidly up to a thousand times in the presence of infection, autoimmune disorders, or tissue injury, peaking within 48 h and declining quickly as the inflammation resolves [21]. Additionally, levels are influenced by gender, age, household income, educational level, smoking status, drinking habits, body weight, physical activity, sleep, and depression [22,23,24].

These biomarkers have shown significance across a broad spectrum of medical conditions such as cardiovascular diseases, diabetes mellitus, chronic pulmonary disorders, various malignancies (including ovarian, cervical, and oral cancers), acute myocardial infarction, psoriasis, ocular inflammatory conditions, and periodontitis [25,26,27,28]. Despite their proven relevance in these pathologies, the application of these biomarkers in implantology remains limited, with most research concentrating on patients with systemic comorbidities or peri-implant inflammatory conditions. Scientific evidence in individuals who are systemically healthy is still scarce, and the predictive value of these biomarkers for early osseointegration outcomes has yet to be confirmed.

Emerging evidence from osteoimmunology suggests that systemic inflammatory activity may influence local bone healing and osseointegration through cytokine-mediated modulation of osteoimmune pathways. Circulating proinflammatory mediators such as interleukin (IL)-1β, IL-6, and tumour necrosis factor-α (TNF-α) can activate the RANK/RANKL/OPG signalling axis, enhancing osteoclastogenesis and promoting bone resorption, while also delaying matrix mineralisation [13,14]. Furthermore, excessive activation of neutrophils and platelets may impair vascular endothelial growth factor (VEGF)-dependent angiogenesis and reduce mesenchymal stem cell recruitment at the implant–bone interface, both of which are critical for new bone formation and remodelling [15,16]. These mechanisms illustrate how even subclinical systemic inflammatory activity could disturb the delicate balance between osteoblast-mediated bone formation and osteoclast-mediated resorption, ultimately affecting the early biological events essential for implant integration. Based on this rationale and the existing literature, we raised the question of whether these systemic biomarkers could predict postoperative outcomes in systemically healthy patients receiving dental implants, thereby supporting the development of simple and cost-effective screening tools for identifying at-risk individuals and optimising pre-surgical planning. To address this, we formulated the null hypothesis that baseline values of NLR, PLR, SII, and CRP would not differ between patients with proper implant osseointegration and those with inadequate outcomes. As an alternative hypothesis, we expected that higher baseline values of these systemic inflammatory biomarkers would be associated with an increased risk of impaired osseointegration, reflecting the potential influence of subclinical systemic inflammation on early wound healing.

## 2. Materials and Methods

### 2.1. Research Design

Patient demographic and health status data from adult patients who underwent implant treatment were retrospectively collected over a five-year period (January 2020–January 2025) from the records of a private clinic in collaboration with the Faculty of Dentistry at “Grigore T. Popa” University of Medicine and Pharmacy, Iași, Romania. Each patient received between one and four endosseous dental implants, depending on the individual clinical presentation and prosthetic treatment plan. The study protocol received approval from the Ethics Committee of “Grigore T. Popa” University of Medicine and Pharmacy, Iași (registration no. 611/19 June 2025). All procedures complied with the ethical principles of the 1964 Declaration of Helsinki and its later revisions (2013).

### 2.2. Criteria for Patient Selection

**Figure 1:**
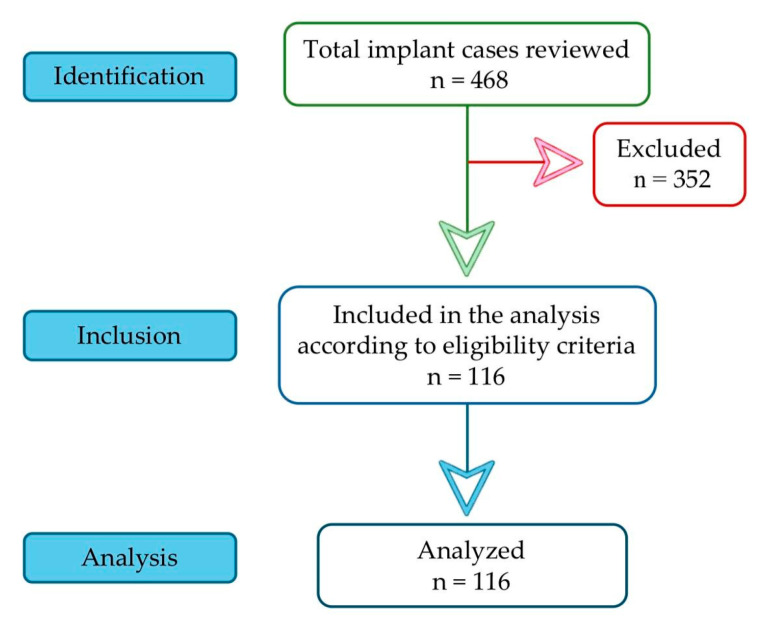
STROBE flowchart.

Among 468 reviewed cases, 116 met the inclusion criteria (Figure 1):

- age: 20–50 years;
- diagnostic records: availability of radiographic (orthopantomography—OPG; cone beam computed tomography CBCT) and photographic documentation before and after treatment;
- periodontal status: either periodontal health or stable periodontal status, defined by a history of periodontitis with <10% bleeding sites and probing depths ≤ 3 mm over the past 6 months [29];
- non-smokers;
- no systemic diseases;
- no history of allergies (including food and metal allergies);
- good treatment adherence and maintenance of satisfactory postoperative oral hygiene;
- ethics: signed informed consent.

The remaining 352 cases were excluded based on the following criteria:

- incomplete or missing clinical documentation (radiographic or photographic records) before or after treatment;
- history of smoking, alcohol dependence, or substance abuse;
- periodontal status matching stage III or IV;
- history of periodontitis treatment within the past six months;
- presence of critical anatomical limitations requiring sinus lift, bone additions, or immediate postextraction implantation;
- use of antibacterial or anti-inflammatory medication within four weeks prior to blood sample collection;
- systemic diseases or conditions affecting bone metabolism, including uncontrolled diabetes, allergies, coronary heart disease, pulmonary disease, malignant tumours, osteoporosis, or ongoing bisphosphonate therapy.

**Figure 2:**
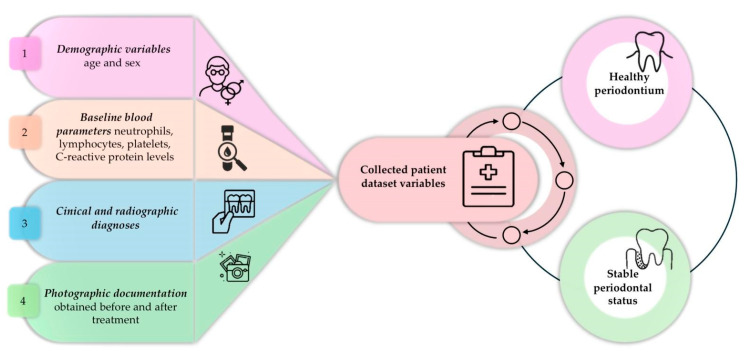
Collected variables and the two groups defined by periodontal status.

Subsequently, patients were divided into two groups based on periodontal status, and the extracted data from their records are summarised in Figure 2.

### 2.3. Operative Technique and Postoperative Care

Fasting blood tests were taken within 24 h before surgery. Patients received amoxicillin-clavulanic acid (2 g) one hour before surgery (Augmentin, Glaxo Wellcome Production ZI, Peyennière, 53100 Mayenne, France) and 1 g twice daily for 7 days afterwards. All procedures were carried out by the same oral and maxillofacial surgeon (N.O.). Immediately before surgery, patients rinsed for 1 min with 0.2% chlorhexidine, and a sterile drape was placed to maintain an aseptic field. Local anaesthesia was administered with 4% articaine and epinephrine 1:100,000 (Ubistein, 3M ESPE, Seefeld, Germany).

A mid-crestal incision through keratinised tissue was made with a No. 15C blade; when necessary, one or two vertical releasing incisions were added. Full-thickness mucoperiosteal flaps were elevated to expose cortical bone. Osteotomies were prepared at 800–1000 rpm with irrigation, adjusted to bone density to prevent overheating. The manual implant insertion was generally at a torque of 35 Newton centimetres (Ncm), which provided strong initial stability. Flaps were repositioned without tension and sutured with 5-0 nylon using a horizontal mattress technique and simple interrupted suture to achieve absolute tension-free adaptation.

Postoperative analgesia with paracetamol and ibuprofen was prescribed for three days. An immediate postoperative radiograph was taken for each patient. During recovery, no removable provisional acrylic dentures (“flippers”) were used; in selected cases, an immediate fixed provisional bridge was provided. After three to six months, the CBCT assessed the appearance of the osseointegrated implants and the surrounding bone. 

The healing period was mostly free of complications. Abutment connection was performed 3–6 months after implant placement. Osseointegration was assessed using combined clinical, radiographic, and resonance frequency analysis (RFA) criteria. Clinically, success required the absence of mobility, pain, or inflammation. Implant stability was measured using the Penguin RFA Kit (Integration Diagnostics Sweden AB, Göteborg, Sweden), and implants with implant stability quotient (ISQ) values above 70 were considered stable with minimal micro-mobility, indicating dental implant survival. Radiographic evaluation confirmed continuous bone contact around the implant without radiolucent areas. Implant failures were cases showing radiographic pathology or mobility within 3–6 months that necessitated immediate removal.

### 2.4. Statistical Analysis

Statistical analyses were conducted using IBM SPSS Statistics software, version 26.0 (IBM Corp., Armonk, NY, USA). The sample size was calculated with an online calculator (www.calculator.net; accessed on 23 June 2025), which determined that 112 patients were required to detect a significant difference at a 5% margin of error and a 95% confidence level. The Kolmogorov–Smirnov and Shapiro–Wilk tests assessed the normality of data distributions for each variable within the group. Differences between groups were further examined using the Kruskal–Wallis H test, with post hoc pairwise comparisons performed via the Mann–Whitney U test. Receiver operating characteristic (ROC) curves were also plotted. Statistical significance was set at *p* < 0.05. 

## 3. Results

**Figure 3:**
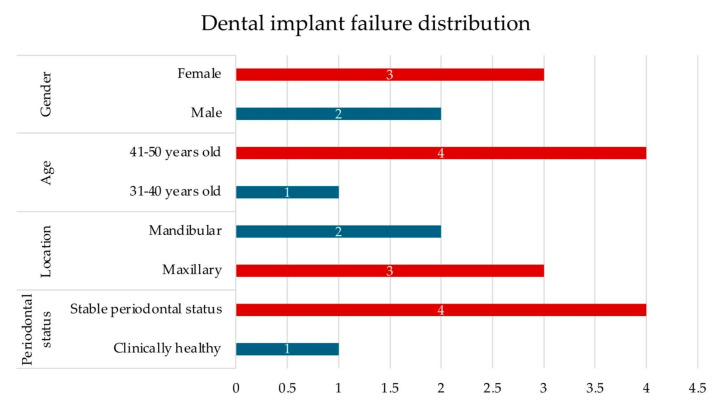
Distribution of dental implant failures by gender, age, location, and periodontal status, expressed as the number of affected patients.

A total of 116 patients were analysed (54 males, 46.6%; 62 females, 53.4%), with a mean age of 38.4 years. The largest group of participants was in the 41–50-year age range (45.7%). Periodontal assessment showed that 49.1% of patients were clinically healthy, while 50.9% demonstrated stable periodontal status. The mean values of systemic inflammatory indices were as follows: CRP 0.85 mg/L, NLR 1.73, PLR 123.5, and SII 463.02. Implant success was achieved in 95.7% of cases, whereas 4.3% experienced failure (Table 1, Figure 3).

Gender- and periodontal status-based comparisons showed differences for PLR *(p =* 0.041, females versus males) and for SII values *(p =* 0.036, clinically healthy patients versus stable periodontal individuals). No other statistically significant associations were observed across age, gender, or periodontal subgroups (Table 2).

**Figure 4:**
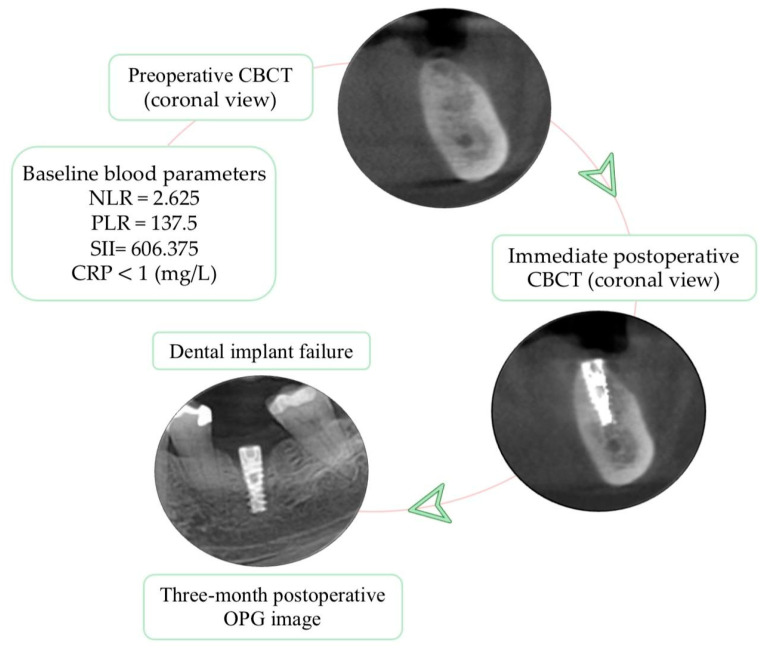
Representative case of early dental implant failure; baseline blood inflammatory biomarkers were within the normal range.

When evaluating the association between baseline blood parameters and implant osseointegration outcomes, no statistically significant differences were observed between neutrophils or lymphocytes. Platelet counts were higher in the dental implant failure group (320.4 versus 260.99 × 10^3^/µL) with a borderline association (*p* = 0.057). Similarly, systemic inflammatory indices, including NLR, PLR, and CRP, showed no significant variation between the two outcome groups. In contrast, SII was significantly higher in failed implants (706.42 versus 452.05; *p* = 0.015) (Table 3; Figure 4).

**Figure 5:**
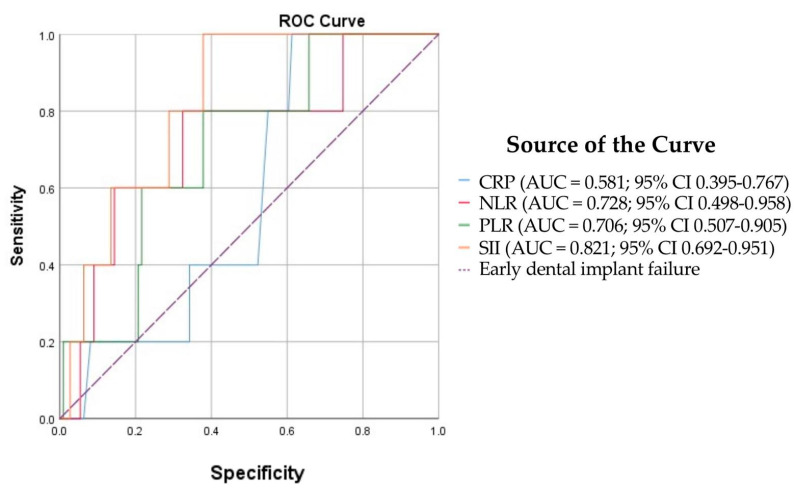
ROC curves for predicting impaired osseointegration in systemically healthy patients.

Using ROC curve analysis, we assessed the predictive capacity of preoperative blood inflammatory biomarkers (CRP, NLR, PLR, SII) for early dental implant failure (impaired osseointegration) in systemically healthy patients, with the resulting predictive performance summarised in Figure 5.

The tested inflammatory biomarkers demonstrated mixed predictive performance for impaired osseointegration (Figure 5):

- SII (AUC = 0.821, SE = 0.066, 95% CI 0.692–0.951, *p* = 0.015) demonstrated good discrimination; the result was statistically significant.
- NLR (AUC = 0.728, SE = 0.117, 95% CI 0.498–0.958, *p* = 0.085) demonstrated fair discrimination, but the result was not statistically significant.
- PLR (AUC = 0.706, SE = 0.102, 95% CI 0.507–0.905, *p* = 0.12) also showed fair discrimination; not significant.
- CRP (AUC = 0.581, SE = 0.095, 95% CI 0.395–0.767, *p* = 0.541) showed poor discrimination; not significant.

## 4. Discussion

This study was driven by clinical observations of implant failure in young, generally healthy patients and the lack of evidence regarding the use of blood inflammatory biomarkers in implantology. Most studies focused on peri-implantitis and evaluated serological markers such as C-reactive protein and cytokines (IL-6, IL-1β, IL-17, tumour necrosis factor-α), along with haematological parameters including neutrophils, haemoglobin, platelets, and lymphocytes [30,31].

This retrospective study of 116 systemically healthy adults examined whether baseline systemic inflammatory biomarkers (CRP, NLR, PLR, and SII) are linked to dental implant outcomes. To minimise confounding factors, we excluded individuals over 50 years old, smokers, and patients with systemic diseases, allergies (including food and metal allergies), stage III/IV periodontitis, or those taking medications that may affect immune function. These patient-related factors can cause immunological imbalance (immune overactivation) and are associated with early implant failures due to unsuccessful osseointegration. In a Swedish cohort of 1875 patients (including smokers and individuals with cardiovascular disease, diabetes, autoimmune disorders, and allergies) treated with implants, seven variables were found to be associated with a higher risk of complications: smoking, exposed implant threads, lack of preoperative antibiotic prophylaxis, greater number of implants, sinus perforations, food allergy, and metal allergy [32].

In our study, the overall success rate of implants was high (95.7%), with only five cases (4.3%) of early dental implant failure. Rates above 90% in healthy adult patients are consistently reported in the literature, including numerous primary studies, validated by systematic reviews and meta-analyses [33,34,35]. Analysing 24,781 implants, with the longest follow-up for a patient exceeding three decades, Jemt [36] found that the risk of failure was highest in the first two years after implant surgery (of 803 total events, 344 occurred in the first year and 131 in the second), and failures were more common at maxillary than mandibular sites. Notably, 58% of patients who experienced failure lost only a single implant.

When categorised by patient characteristics, most biomarkers showed increased levels across different age groups and periodontal subgroups, but these differences were generally not statistically significant. Only two comparisons reached significance: females had higher PLR values than males (mean 132 versus 113; *p* = 0.041), and SII was higher in participants with a stable periodontal status compared with those who were clinically healthy (mean 478 versus 446; *p* = 0.036). Given that “stable” status implies a history of periodontitis with current clinical control, this elevation may reflect residual low-grade immune activation despite clinically acceptable parameters. These patterns align with population-based reference data from the Rotterdam cohort (8711 participants), which reported reference intervals of NLR mean 1.76 (0.83–3.92), PLR mean 120 (61–239), and SII mean 459 (189–1168), showing inflammatory indices increasing with age, with PLR and SII higher in women [20].

Apart from SII, baseline hematologic and inflammatory indices showed no significant differences between properly and impaired osseointegration (all *p* > 0.05). Platelet counts were higher in the failure group (320 versus 260.9 × 10^3^/µL) with borderline significance (*p* = 0.057), while SII was significantly higher in failed implants (706 versus 452; *p* = 0.015), indicating an association with early failure. Higher SII values may reflect a temporary systemic inflammatory response that could influence early bone healing through mechanisms described in osteoimmunology. Given the limited number of implant failures and the absence of longitudinal biomarker evaluation, a definitive causal link between systemic inflammation and osseointegration cannot be established.

The association between the CRP, NLR, PLR, and SII has been identified as providing relevant data regarding periodontal diseases and, moreover, in peri-implantitis. In a meta-analysis of 10 studies (all conducted in Asia), Almășan et al. [26] found that the mean NLR was higher in periodontitis than in healthy controls by 0.41 (95% CI, 0.12–0.70; *p* = 0.006). For PLR, the mean difference was 1.83 (95% CI, −9.38 to 13.04), which was not statistically significant. In their systematic review, the authors concluded that blood cell–derived ratios are associated with periodontitis, suggesting utility as emerging inflammatory biomarkers for disease systemic links and prognosis/grading; however, they cautioned that methodological limitations of the included studies warrant prudent interpretation.

In our study, the mean CRP levels were 0.87 mg/L in the stable periodontal status group, 0.83 mg/L in controls, and 0.96 mg/L in the implant-failure group, differences that did not reach statistical significance.

It is plausible that the mechanism of CRP induction is similar in peri-implantitis and periodontitis, with potential systemic implications. Across clinical studies using serum CRP in patients with a history of periodontitis, evidence indicates a dose–response relationship with disease severity (higher CRP in more severe periodontitis) and potential reversibility after therapy (several reports describe reductions in serum CRP following periodontal treatment) [22,23]. In a clinic-based cross-sectional study, participants with periodontitis had greater odds of altered CRP (≥3 mg/L) than those without the disease (OR = 3.27; 95% CI 1.42–7.52). After adjustment for age, smoking, and BMI, CRP levels were 1.7 times higher in individuals with periodontitis, indicating the association persists beyond key confounders. Obesity also amplified CRP (adjusted ratio 3.48 versus normal weight), underlining the importance of controlling adiposity when interpreting CRP and periodontitis links [24]. 

Although evidence remains limited, peri-implantitis seems connected to systemic inflammation. After an initial non-significant CRP signal [37], subsequent studies reported elevated serum CRP levels, and two studies observed reductions following local therapy [31]. In a case–control study with 100 participants, the authors found that serum CRP was significantly higher in patients with peri-implantitis (mean 0.615 mg/dL) compared to systemically healthy controls (mean 0.201 mg/dL), based on fasting morning venous samples analysed by latex-enhanced nephelometry [38]. Similarly, Khichy et al. [39] reported in a study involving 40 patients (20 with peri-implantitis; 20 controls) higher CRP (0.795 versus 0.294 mg/dL) and IL-6 (12.178 versus 6.458 pg/mL) levels in peri-implantitis. Clinically, these findings suggest that CRP may act as an additional systemic marker reflecting peri-implant inflammatory burden; however, the study’s case–control design and sample size indicate that longitudinal research is necessary to establish its prognostic value and response to treatment.

Based on the study’s results, only SII was significantly higher in patients with impaired outcomes; overall, the evidence was insufficient to reject the null hypothesis, which was therefore retained.

The findings should be interpreted cautiously due to several limitations: a retrospective, single-centre design; strict inclusion criteria; a small number of early implant failures; reliance on a single time-point biomarker assessment; and the lack of modelling for key procedural variables, such as jaw/site, bone quality, implant surface, torque, loading, and surgeon. Overall, these constraints indicate that the results are preliminary and need confirmation through larger, prospective, multicentre studies that include longitudinal monitoring of systemic inflammatory biomarkers and comprehensive adjustment for patient, site, and procedural factors.

## 5. Conclusions

Clinically, in systemically healthy patients, routine preoperative screening based solely on blood inflammatory biomarkers, NLR, PLR, and CRP, did not enhance the assessment of early implant failure risk. Conversely, SII demonstrated good, statistically significant discriminatory ability and may have supplementary value. Notably, mean biomarker levels remained within reference ranges, a detail that could be overlooked during routine laboratory interpretation. Well-designed prospective studies are required to validate SII and clarify the predictive utility of blood biomarkers for early implant failure.

## Figures and Tables

**Table 1:** Characteristics of study participants (N = 116).

	Patient Characteristics	Count (N)	Percentage (%)	Tests of Normality	
				Kolmogorov–Smirnov	Shapiro–Wilk
Variable	*Gender*			*p* = 0.000	*p* = 0.000
	Male	54	46.6		
	Female	62	53.4		
	*Age*			*p* = 0.000	*p* 0.000
	20–30 years old	22	19.0		
	31–40 years old	41	35.3		
	41–50 years old	53	45.7		
	Mean ± SD (Minimum−Maximum)			38.42 ± 8.255 (20–50)	
	*Periodontal status*			*p* = 0.000	*p* = 0.000
	Clinically healthy	57	49.1		
	Stable periodontal status	59	50.9		
	*Neutrophil level* (10^3^/µL)			*p* = 0.007	*p* = 0.000
	Mean ± SD (Minimum−Maximum)			3.80 ± 1.41 (1.24–11.08)	
	Reference range: 2–8 ×10^3^/µL				
	*Lymphocyte level* (10^3^/µL)			*p* = 0.085	*p* = 0.030
	Mean ± SD (Minimum−Maximum)			2.33 ± 0.71 (0.90–4.48)	
	Reference range: 1–4 ×10^3^/µL				
	*Platelet level* (10^3^/µL)			*p* = 0.008	*p* = 0.000
	Mean ± SD (Minimum−Maximum)			263.55 ± 58.91 (162–454)	
	Reference range: 150–450 ×10^3^/µL				
	*C-reactive protein level* (mg/L)			*p* = 0.011	*p* = 0.000
	Mean ± SD (Minimum−Maximum)			0.85 ± 0.54 (0.02–2.7)	
	Reference range: 0–5 mg/L				
	*Neutrophil-to-lymphocyte ratio*			*p* = 0.000	*p* = 0.000
	Mean ± SD (Minimum−Maximum)			1.73 ± 0.75 (0.52−6)	
	*Platelet-to-lymphocyte ratio*			*p* = 0.000	*p* = 0.000
	Mean ± SD (Minimum−Maximum)			123.50 ± 51.62 (54.73–328.88)	
	*Systemic immune-inflammatory index*			*p* = 0.000	*p* = 0.000
	Mean ± SD (Minimum−Maximum)			463.02 ± 258.74 (127.12–1776)	
	*Postoperative outcomes*			*p* = 0.000	*p* = 0.000
	Dental implant survival Proper implant osseointegration	111	95.7		
	Early dental implant failure Inadequate implant osseointegration	5	4.3		

SD–standard deviation.

**Table 2:** Association between gender, age and periodontal status and systemic inflammatory biomarkers.

	Age (Mean ± SD)			Gender (Mean ± SD)		Periodontal Status (Mean ± SD)	
	20–30 Years Old	31–40 Years Old	41–50 Years Old	Male	Female	Healthy	Stable
N 10^3^/µL	3.88 ± 1.29	3.70 ± 1.09	3.84 ± 1.67	3.77 ± 1.45	3.83 ± 1.39	3.64 ± 1.08	3.93 ± 1.67
	Kruskal–Wallis Test; *p* = 0.756			Mann–Whitney test; *p* = 0.897		Mann–Whitney test; *p* = 0.522	
L 10^3^/µL	2.55 ± 0.89	2.24 ± 0.64	2.31 ± 0.68	2.42 ± 0.71	2.26 ± 0.71	2.34 ± 0.73	2.33 ± 0.70
	Kruskal–Wallis Test; *p* = 0.438			Mann–Whitney test; *p* = 0.141		Mann–Whitney test; *p* = 0.667	
PLT 10^3^/µL	251.68 ± 52.17	260.49 ± 62.22	270.84 ± 58.96	253.05 ± 54.35	272.69 ± 61.58	254.21 ± 52.55	272.57 ± 63.60
	Kruskal–Wallis Test; *p* = 0.461			Mann–Whitney test; *p* = 0.055		Mann–Whitney test; *p* = 0.126	
NLR	1.70 ± 1.06	1.74 ± 0.66	1.73 ± 0.67	1.66 ± 0.80	1.79 ± 0.70	1.70 ± 0.87	1.75 ± 0.62
	Kruskal–Wallis Test; *p* = 0.539			Mann–Whitney test; *p* = 0.161		Mann–Whitney test; *p* = 0.228	
PLR	111.51 ± 55.86	127.56 ± 59.61	125.33 ± 42.70	113.16 ± 43.72	132.50 ± 56.45	122.54 ± 62.16	124.42 ± 39.39
	Kruskal–Wallis Test; *p* = 0.193			Mann–Whitney test; *p* = 0.041 *		Mann–Whitney test; *p* = 0.105	
SII	436.37 ± 322.80	462.73 ± 240.85	474.29 ± 246.82	420.59 ± 243.31	499.97 ± 267.95	446.76 ± 293.13	478.72 ± 221.96
	Kruskal–Wallis Test; *p* = 0.410			Mann–Whitney test; *p* = 0.058		Mann–Whitney test; *p* = 0.036 *	
CRP mg/L	0.98 ± 0.51	0.80 ± 0.60	0.84 ± 0.50	0.81 ± 0.48	0.89 ± 0.59	0.83 ± 0.52	0.87 ± 0.57
	Kruskal–Wallis Test; *p* = 0.303			Mann–Whitney test; *p* = 0.059		Mann–Whitney test; *p* = 0.866	

* *p* < 0.05; SD—standard deviation; N—neutrophil level; L—lymphocyte level; PLT—platelet level; NLR—neutrophil-to-lymphocyte ratio; PLR—platelet-to-lymphocyte ratio; SII—systemic immune-inflammatory index; CRP—C-reactive protein level.

**Table 3:** Association between baseline blood parameters to implant osseointegration.

Baseline Clinical Parameters	Dental Implant Survival			Dental Implant Failure		
	Mean ± SD	Minimum	Maximum	Mean ± SD	Minimum	Maximum
N 10^3^/µL	3.77 ± 1.43	1.24	11.08	4.37± 0.49	3.8	5.1
	Mann–Whitney test, U = 404.000, *p* = 0.085					
L 10^3^/µL	2.34 ± 0.71	0.90	4.48	2.19± 0.84	1.30	3.54
	Mann–Whitney test, U = 234.500, *p* = 0.559					
PLT 10^3^/µL	260.99 ± 57.26	162	454	320.4± 73.25	231	427
	Mann–Whitney test, U = 417.500, *p* = 0.057					
NLR	1.71 ± 0.75	0.52	6	2.18 ± 0.66	1.27	2.92
	Mann–Whitney test, U = 404.000, *p* = 0.085					
PLR	121.57 ± 48.87	54.73	328.88	166.38 ± 92.41	97.17	328.46
	Mann–Whitney test, U = 392.000, *p* = 0.12					
SII	452.05 ± 251.73	127.12	1776	706.42 ± 323.66	439.23	1248.15
	Mann–Whitney test, U = 456.000, *p* = 0.015 *					
CRP mg/L	0.85 ± 0.55	0.02	2.70	0.96 ± 0.44	0.6	1.72
	Mann–Whitney test, U = 322.500, *p* = 0.541					

* *p* < 0.05; SD—standard deviation; N—neutrophil level; L—lymphocyte level; PLT—platelet level; NLR—neutrophil-to-lymphocyte ratio; PLR—platelet-to-lymphocyte ratio; SII—systemic immune-inflammatory index; CRP—C-reactive protein level.

## Data Availability

The data that support the findings of this study are available on request from the corresponding author.

## Abbreviations

The following abbreviations are used in this manuscript:

CBCT	Cone beam computed tomography
CRP	C-reactive protein level
L	Lymphocyte level
N	Neutrophil level
NLR	Neutrophil-to-lymphocyte ratio
OPG	Orthopantomography
PLR	Platelet-to-lymphocyte ratio
PLT	Platelet level
SD	Standard deviation
SII	Systemic immune-inflammatory index
